# Beta-Endorphin and Oxytocin in Patients with Alcohol Use Disorder and Comorbid Depression

**DOI:** 10.3390/jcm10235696

**Published:** 2021-12-03

**Authors:** Olga V. Roschina, Lyudmila A. Levchuk, Anastasiia S. Boiko, Ekaterina V. Michalitskaya, Elena V. Epimakhova, Innokentiy S. Losenkov, German G. Simutkin, Anton J. M. Loonen, Nikolay A. Bokhan, Svetlana A. Ivanova

**Affiliations:** 1 Mental Health Research Institute, Tomsk National Research Medical Center of the Russian Academy of Sciences, 634014 Tomsk, Russia; roshchinaov@vtomske.ru (O.V.R.); rla2003@list.ru (L.A.L.); anastasya-iv@yandex.ru (A.S.B.); uzen63@mail.ru (E.V.M.); ElenaZhernova@sibmail.com (E.V.E.); innokenty86@mail.ru (I.S.L.); ggsimutkin@gmail.com (G.G.S.); nikolay.bokhan.tomsk.russia@gmail.com (N.A.B.); ivanovaniipz@gmail.com (S.A.I.); 2 Groningen Research Institute of Pharmacy (GRIP), PharmacoTherapy, -Epidemiology & -Economics, University of Groningen, 9713AV Groningen, The Netherlands; 3 Psychiatry, Addictology and Psychotherapy Department, Siberian State Medical University, 634050 Tomsk, Russia

**Keywords:** β-endorphin, oxytocin, blood, alcohol use disorder, addiction-related mood change, aggressive behavior

## Abstract

Background: The neuropeptides β-endorphin and oxytocin are released into the bloodstream as hormones from the pituitary gland but also have an important function as neuroregulators in the forebrain. The blood levels of both polypeptides have been shown to reflect depressive symptoms. β-Endorphin, in particular, is also involved in abstinence from alcohol. Methods: The serum levels of β-endorphin and oxytocin were measured during the early withdrawal phase in patients with alcohol use disorder (AUD) with (*N* = 35) or without (*N* = 45) depressive comorbidity and compared with those in healthy volunteers (*N* = 23). In addition to comparing the groups, the study examined whether serum levels correlated with various psychometric measures of dependence, depression and aggression, as well as with clinical characteristics of dependence. Results: Both serum levels of beta-endorphin and oxytocin were significantly lower in patients than those in healthy controls (*p* = 0.011 for β-endorphin and *p* = 0.005 for oxytocin, Kruskal–Wallis test). In patients with depressive comorbidity, the significance was greatest (*p* = 0.005 for β-endorphin and *p* = 0.004 for oxytocin, U-test). There was no correlation with clinical or psychometric parameters (*p* > 0.05, Spearman test), but beta-endorphin levels did correlate significantly with physical aggression (*p* = 0.026, Spearman test). Conclusions: Serum levels of β-endorphin and oxytocin are lower in patients with AUD, particularly in those with depressive comorbidity. β-Endorphin levels correlated with physical aggression according to the Buss–Durkee (BDHI) estimates.

## 1. Introduction

β-Endorphin is one of the neuropeptides with both neuronal and endocrine localization and function [1]. It is one of the opioid peptides and arises from pro-opiomelanocortin (POMC) [1,2], which also produces adrenocorticotropic hormone (ACTH), β-lipotropin (β-LPH) and, as another component of the latter, β-melanocyte-stimulating hormone (β-MSH). β-Endorphin is a 31-amino acid polypeptide which binds to μ- and δ-opioid receptors, but the peptides derived from the other part of POMC bind to specific melanocortin receptors [3]. Peptides from the POMC precursor are localized in corticotrophs of the anterior and in melanotrophs of the intermediate lobe of the pituitary gland [4,5]. They have also been identified in the perikarya of the arcuate nucleus of the basal hypothalamus as well as the nucleus of the solitary tract in the brainstem, and these neurons’ extensions terminate in diverse brain regions. Nerve cells of the arcuate nucleus expressing POMC play an important role in energy homeostasis, but this is via melanocortin receptors [6]. Release of β-endorphin in the arcuate nucleus is induced by exposure to pain and anxiety [7], but the ultimate role of this peptide is unknown. From the anterior pituitary, but not from the intermediate lobe, ACTH and the peptides derived from β-LPH (such as β-endorphin) are secreted simultaneously [4]. ACTH, MSH and β-endorphin fragments have pronounced behavioral effects, so the specific role of β-endorphin is difficult to elucidate. In spite of this uncertainty, a role was suggested in the manifestation of some psychiatric diseases [8,9]. 

Oxytocin is another neuropeptide secreted by the pituitary gland, in this case the posterior pituitary or neurohypophysis. It is phylogenetically considerably older than POMC; it previously played a role as a neurotransmitter in very early animals—similar to sea anemones, corals, *Hydra*, jellyfish—that populated the earth more than 700 million years ago [10,11]. The genes for the opioid peptides—including those for POMC—are of a far later date and originated in jawless vertebrates and their direct ancestors [12,13]. Oxytocin is a cyclic nonapeptide synthesized in the paraventricular nucleus (PVN) and supraoptic nucleus (SON) of the hypothalamus and released by the posterior pituitary into peripheral circulation. It differs in two places during amino acid composition from vasopressin, which has the same peptide as an ancestor during evolution [14]. It was previously thought that most hypothalamic oxytocin was produced for secretion in the neurohypophysis with only limited intracerebral connectivity of oxytocin-containing neurons remaining within the hypothalamus or going to the brainstem. New techniques have shown that oxytocin is widely present throughout the forebrain and is released from many neuronal elements in a paracrine manner and at synapses [15,16]. Oxytocin is best known for its role in lactation and partus, but it has many other effects in the periphery, as well as particularly in the central nervous system [17,18,19,20,21]. This includes an important role for oxytocin in addiction to alcohol (and other drugs) [22,23,24,25,26], supporting theories invariably paying close attention to the interaction of oxytocin with dopaminergic neurotransmission [22,23,26]. However, we would like to point out that the role of dopamine in the mechanism of addiction is less certain, partly because oxytocin has recently been shown to decrease alcohol drinking in alcohol dependence, but not in nondependent drinking when dopamine may have a more prominent role [27].

We investigated the parallel secretion of β-endorphin and oxytocin in the peripheral circulation in recently admitted patients with alcohol use disorder (AUD) with and without concomitant depressive disorder. We paid attention to the possible relationship with some of the clinical and mental characteristics of addiction and associated depressive comorbidity. 

## 2. Materials and Methods

### 2.1. Design

In a cross-sectional transdiagnostic prospective cohort study, the relationship between phenotype measures of depression and alcohol use disorder (AUD) was compared across two diagnostic groups (AUD with and without comorbid mood disorder (MD)) and age-matched healthy controls. The study was carried out in accordance with the Code of Ethics of the World Medical Association (Declaration of Helsinki 1975, revised in Fortaleza, Brazil, 2013) and approved by the Institutional Medical Review Board (Protocol of the Local Ethics Committee at the Research Institute of Mental Health No. 101 from 13 June 2017). All participants provided written informed consent.

### 2.2. Participants

Participants with AUD with comorbid MD (*n* = 35) and without any comorbidity (*n* = 45) were recruited from the departments of affective and addictive states of the Mental Health Research Institute (MHRI) of the Tomsk National Research Medical Center. The inclusion criteria were: a diagnosis of AUD (F10.2) with or without depressive episode/dysthymia (F31, F32, F33, F34.1) according to ICD-10 [28]; ages 18–60 years. We excluded patients with other comorbid mental disorders, for instance schizophrenia, intellectual disability and alcoholic psychoses, as well as patients with acute physical diseases. The screening for relevant pathology for in/exclusion of subjects was performed through clinical assessment by three trained psychiatrists on the first day of admission. Alcohol history and withdrawal severity were assessed correspondingly. The control group consisted of 23 healthy volunteers recruited through local advertisements at the MHRI and Tomsk State University. Healthy individuals were screened using a self-report questionnaire. The questionnaire screens for both physical and mental pathology, e.g., endocrine, neurological, gynecological and psychiatric disorders.

As shown in Table 1, the studied patient groups were comparable with respect to age and sex.

Comorbid Affective Disorder in the AUD-MD group was defined by the presence of depressive episodes of mild to moderate severity in context of Bipolar Affective Disorder F31 (20%, *n* = 7), Depressive Episode F32 (17.1%, *n* = 6), Recurrent Depressive Disorder F33 (28.6%, *n* = 10) or Dysthymia (34.3%, *n* = 12) (*p* = 0.457, chi-square test).

Patients in a state of withdrawal received benzodiazepine therapy to alleviate withdrawal symptoms. The duration of alcohol withdrawal as estimated by the treating psychiatrist was on average 3 days after admission (AUD group 3 (3; 4.5) days, AUD-MD group 3 (2; 4) days (*p* = 0.28, U-test). After relief of withdrawal symptoms, patients take specific psychotropic therapy with antidepressants of various groups (AUD group 62.2% (*n* = 28), AUD-MD group 65.7% (*n* = 23)), anticonvulsants (AUD group 31.1% (*n* = 14), AUD-MD group 31.4% (*n* = 11)) or other drugs (AUD group 6.7% (*n* = 3), AUD-MD group 2.9% (*n* = 1)).

### 2.3. Measurements

#### 2.3.1. Phenotype Measures

Depressive symptoms were assessed using the 17-item Hamilton Depression Rating Scale (HAMD-17) as derived from the Structured Interview Guide for the Hamilton Depression Rating Scale—Seasonal Affective Disorder version (SIGH-SAD) [29,30]. The other transdiagnostic symptom domains were assessed using Russian translations of commonly applied self-report questionnaires [31]. All self-report questionnaires were common Russian translations of the originals (verified by back translation into English). Anhedonia was assessed by applying the Snaith–Hamilton Pleasure Scale (SHAPS) [32]. Alcohol use and dependency were measured using the Alcohol Use Disorders Identification Test-Consumption (AUDIT-C) [33]. Craving was assessed using the Obsessive Compulsive Drinking Scale (OCDS) [34]. The psychometric tests were performed during the first week of admission, after remission of withdrawal or intoxication symptoms. Leading patterns of hostility and aggressive reactions of patients and healthy volunteers were detected with the Buss–Durkee Hostility Inventory (BDHI) [35]. The BDHI is a self-reporting questionnaire containing 75 points to estimate seven aspects of hostility: assault, indirect hostility, irritability, negativism, resentment, suspicion, and verbal hostility. Details on the other scales have been described previously [31].

#### 2.3.2. Biomarkers

Peripheral venous blood was collected from each subject between 8:00 and 9:00 a.m. on the morning after hospital admission, after eight hours of overnight fasting before the intake of any food or medication. Blood was sampled in BD Vacutainer tubes with a coagulation activator and centrifuged at 2000 RCF at 4 °C for 20 min. Serum samples were stored at −80 ℃ until they could be analyzed.

Concentrations of analytes were determined on the MAGPIX multiplex analyzer (Luminex, Austin, TX, USA) using xMAP^®^ Technology. Panel HNPMAG-35K by MILLIPLEX^®^ MAP (Merck, Darmstadt, Germany) was used to determine the levels of the markers β-endorphin and oxytocin. The detected information was processed by special Luminex xPONENT^®^ software with subsequent export of data to the MILLIPLEX^®^ Analyst 5.1 program. 

### 2.4. Data Analysis

The normal distribution of the values of the variables was checked using the Shapiro–Wilk test. The Kruskal–Wallis test was used to compare the β-endorphin and oxytocin content in the serum of the groups, followed by pairwise posteriori comparisons using the Mann–Whitney test. Identification of the most effective diagnostic markers in the studied patient groups was carried out using ROC analysis. All analyses were carried out using SPSS version 26 Windows.

## 3. Results

Patients in both research groups were comparable for the duration of AUD: in the pure AUD group, median lasting alcohol abuse was 14.5 (8; 20) years (lower quartile, upper quartile); in the AUD-MD group, it was 13 (8; 19) years (*p* = 0.710, U-test). In the case of comorbidity, MD developed 7 (2; 13) years ago. Despite the same duration of a disease and a comparable number of heavy drinking days (AUD group 12 (5; 30) days, AUD-MD group 7 (5; 14) days (*p* = 0.109, U-test)), patients with pure AUD were more tolerant to alcohol effects: the tolerance in the AUD group was 17 (11; 29.5) standard drinks, yet in the AUD-MD group it was 11 (11; 18) drinks (*p* = 0.032, U-test).

Comparison of clinical psychometric assessment did not yield any differences in estimates of alcohol abuse intensiveness, craving or anhedonia level (*p* > 0.05, U-test), while demonstrating significant differences in depressive symptoms (*p* < 0.001, U-test) (Table 2).

Further, the research of aggressive and hostile reactions was provided by the Buss–Durkee Hostility Inventory (BDHI) in the study groups and healthy controls. As can be seen from Table 3, aggressive and hostile reactions of the AUD-MD comorbidity patients and the pure MD groups significantly differ from the corresponding forms of mentally healthy individuals.

The main results of peripheral markers found in patients with AUD and AUD-MD comorbidity are presented in Table 4.

Comparing the β-endorphin serum concentration in patients and healthy controls demonstrated statistically significant differences between the three groups (*p* = 0.011). Pairwise comparisons of the study groups revealed that the β-endorphin content in the blood serum of patients with an AUD-MD comorbidity is statistically significantly lower than this indicator in mentally healthy individuals (*p* = 0.005). Comparison of the serum oxytocin content also revealed statistically significant differences between the studied patients and healthy individuals (*p* = 0.005). Blood serum oxytocin concentration in the AUD-MD patients significantly differed from the corresponding parameter in mentally healthy individuals and patients with pure AUD (*p* = 0.004 and *p* = 0.017, respectively). Differences in the β-endorphin serum level in patients with AUD and controls or the AUD and AUD-MD groups did not reach the level of statistical significance (*p* = 0.065 and *p* = 0.080, respectively

The influence of β-endorphin and oxytocin markers on the development of addictive disorders was determined by ROC analysis. Provided ROC analysis indicates the participation of β-endorphin and oxytocin in the development of comorbidity of AUD and MD (AUC = 0.714; 95%Cl 0.562–0.866; *p* = 0.007 and AUC = 0.717; 95%Cl 0.566–0.867; *p* = 0.007, correspondingly).

Searching for correlations according to Spearman between blood levels of the bio-markers and clinical characteristics (such as illness and withdrawal duration, alcohol tolerance and number of heavy drinking days), as well as the obtained psychometric data (HDRS-17, AUDIT-C, OCDC and SHAPS score), found no statistically significant interrelationships (Table 5).

Correlation analysis of the relationship between aggressive and hostile reactions with β-endorphin and oxytocin revealed a negative correlation between physical aggression and β-endorphin in the AUD-MD patients (r = −0.376; *p* = 0.049) (Table 6).

## 4. Discussion

In this study, we measured the serum concentrations of β-endorphin and oxytocin in a group of newly admitted patients with alcohol use disorder (*n* = 80) who did (*n* = 35) or did not (*n* = 45) also suffer from depression, and compared them with those in healthy volunteers (*n* = 23). We compared the results with data from measurements of the various clinical features of AUD and MD, as well as with expressions of aggression as measured by the BDHI. Both levels were significantly lower in the people with AUD than in the healthy volunteers, especially in those who also suffered from depression. Patients with AUD also showed more aggression than the healthy controls according to numerous sub-items of the BDHI, and, again, this was especially the case in people with depressive comorbidity. Only β-endorphin showed a significant negative correlation in the group of patients with comorbid depression, and then only with one of the sub-items of the BDHI: physical aggression. None of the clinical features studied correlated with the neuropeptide serum levels in patients with AUD, either with or without depression.

This is certainly not the first time that blood levels of β-endorphin or oxytocin have been measured in people with alcoholism or a depressive disorder. However, we found only two studies in which both beta-endorphin and oxytocin were measured in the same patients [36,37]. Marchesi and colleagues [36] measured levels of oxytocin, vasopressin, estrone and β-endorphin during 28 days of detoxification in 13 men with AUD and compared them with nine age- and sex-matched normal controls. During this entire period, oxytocin levels were consistently higher, and β-endorphin levels lower, than in healthy volunteers. Haass-Koffler and others [37] investigated the relationship between serum levels of oxytocin, β-endorphin, melatonin, α-melanocyte-stimulating hormone, substance P and orexin with indicators of alcohol and tobacco use in 19 people (46% men or women). They found a positive correlation with the objective measure of smoking only for oxytocin, β-endorphin and orexin. Chronic alcohol use and subsequent abstinence is associated with a stress response [38], and, due to the occurrence of ACTH and β-endorphins in the same precursor molecule POMC, a parallel secretion from the pituitary gland is to be expected. This parallel secretion has been the subject of much research, especially in the context of escaping suppression after administering a low dose of dexamethasone in severe depression [39,40,41,42,43]. However, in the case of alcohol abuse, there seems to be more to it than that. In chronic alcohol consumption, the baseline levels of β-endorphin [44,45,46,47] and the response of ACTH and β-endorphin to stress/anxiety, the CRF and opioid receptor antagonists, appear to be lower than those in healthy volunteers [47,48,49]. This probably has a heritable background, as people from high-risk families for alcoholism have a lower β-endorphin response to acute alcohol administration than healthy volunteers [46,50]. 

It is somewhat surprising that the influence of alcohol on the blood levels of oxytocin has been studied so little and with mixed results [36,37,51,52]. It has long been known that oxytocin has strong behavioral effects that could play a role in alcoholism [25,26]. In the best of the previous studies, Lenz and colleagues [52] found significantly elevated oxytocin levels in early abstinence from alcohol in patients with AUD, more so in men than in women and also dependent on whether or not they smoked. The blood levels of oxytocin we found are lower in early abstinence of the AUD patients than in the healthy controls, especially in those with depressive comorbidity. Oxytocin has often been suggested to play an important role in the development of depression (and mania) [53,54,55,56,57,58,59,60,61,62], especially post-partum depression [63,64], and also in the development of ‘aggressive’ behavioral disorders [65,66,67,68]. This may be related to the influence of oxytocin on certain forms of social behavior [17,54,55]. We saw no relationship of oxytocin concentrations with the clinical features of alcoholism and depression, nor with expressions of aggression.

In interpreting our study, we encountered several limitations. Due to the different (albeit insignificant) gender composition of the relatively small study groups, the sex of the participants may have influenced the results. However, we included all suitable patients as admitted to our clinic without selecting or excluding by sex. Therefore, the differences in composition may also be related to the effect of biological factors. Moreover, we only measured serum levels of these hormones and cannot be certain whether these correlate with effects as a neuroregulatory entity within the brain.

## 5. Conclusions

Serum levels of β-endorphin and oxytocin are lower in patients with AUD, particularly in those with depressive comorbidity. The levels did not correlate with clinical nor psychometric measures, with the exception of the β-endorphin levels, which correlated with physical aggression according to the Buss–Durkee (BDHI) estimates.

## Figures and Tables

**Table 1:** Age and sex composition of the studied groups (median (lower quartile; upper quartile); Me (Q1; Q3)).

Index	AUD Patients (*n* = 45)	AUD-MD Comorbidity (*n* = 35)	Control Group (*n* = 23)	Statistical Significance
Age (years)	45 (40; 54)	44 (36.75; 48.25)	40 (25; 50)	*p* = 0.166, Kruskal–Wallis test
Sex (Female/Male)	18.6%/81.4%	35.3%/64.7%	47.8%/52.5%	*p* = 0.065, chi-squared test

**Table 2:** Main clinical–psychometric characteristics in persons of the two patient groups (Me (Q1–Q3)).

Clinical Scale	AUD Group	AUD-MD Group	*p* (U-Test)
HAMD-17	11 (6.5; 15)	21 (18; 27)	0.001
AUDIT-C	30 (23; 34)	21 (18; 27)	0.171
OCDC	35 (30; 45)	35 (22; 42)	0.440
SHAPS	1 (0; 3)	1 (0; 3)	0.922

HAMD-17—Hamilton Depression Rating Scale; AUDIT-C—Alcohol Use Disorders Identification Test-Consumption; OCDC—Obsessive Compulsive Drinking Scale; SHAPS—Snaith-Hamilton Pleasure Scale.

**Table 3:** Forms of aggressive and hostile reactions by the BDHI in persons of the two patient groups and in healthy controls (Me (Q1–Q3)).

Buss–Durkee Index	AUD Patients	AUD-MD Patients	Controls	*p* (Kruskal–Wallis Test) ¶
Physical aggression (assault)	4 (3–8) **# *p* = 0.023**	7 (5–9) * *p* = 0.023	4.5 (3–7)	**0.028**
Indirect hostility	4 (3–6)	5 (4–6)	4 (2.75–4.25)	0.161
Irritability	5 (3–7)	6 (4.25-7) * *p* = 0.015	4 (2.75–4.25)	0.06
Negativism	3 (2–3)	2 (1–3)	1.5 (1–3)	0.183
Resentment	5 (2–6) * *p* = 0.035	5 (3.25-6) * *p* = 0.011	2.5 (1–4)	**0.048**
Suspicion	5 (4–7) * *p* = 0.001	5 (3.25–7.75) * *p* = 0.004	2 (2–4)	**0.004**
Verbal hostility	7 (4–10)	7.5 (5.25-9)	7 (4–7.25)	0.349
Guilt	7 (5–8) * *p* = 0.016	7 (5–8) * *p* = 0.006	4 (2.5–6.25)	**0.018**
Aggressiveness Index	21 (17–28) **# *p* = 0.039**	24 (21–28.75)	18 (14–23)	**0.047**
Hostility Index	10 (6–13) * *p* = 0.003	9 (7.25–11.75) * *p* = 0.003	4.5 (3–7.75)	**0.006**

Note: * and in **bold**—significantly different in comparison with mentally healthy individuals to the Mann–Whitney test. # and in **bold**—significantly different in comparison with AUD-MD comorbidity to the Mann–Whitney test. ¶ and in **bold**—significantly different in comparison between the groups of patients with AUD, patients with AUD-MD and healthy controls.

**Table 4:** Serum concentration as measured in the two patient groups and in healthy individuals (Me (Q1–Q3)).

Biological Marker	AUD Patients (*n* = 45)	AUD-MD Patients (*n* = 35)	Control Group (*n* = 23)	*p* (Kruskal–Wallis Test) ¶
β-endorphin	295.71 (220.36–413.5)	239.29 (180.42–306.04) * *p* = 0.005	368.22 (215.98–595.21)	**0.011**
Oxytocin	180.24 (112.55–267.13) **# *p* = 0.017**	135.19 (90.27–195.56) * *p* = 0.004	265.23 (111.73–499.66)	**0.005**

Note: * and in **bold**—significantly different in comparison with mentally healthy individuals to the Mann–Whitney test. # and in **bold**—significantly different in comparison with AUD-MD comorbidity to Mann–Whitney test. ¶ and in **bold**—significantly different in comparison between the groups of patients with AUD, patients with AUD-MD and healthy controls.

**Table 5:** Correlation analysis by Spearman between the β-endorphin and oxytocin level and main clinical-psychometric characteristics in the two patient groups.

Research Criteria	AUD Group		AUD-MD Group	
	β-Endorphin	Oxytocin	β-Endorphin	Oxytocin
MD duration			r = −0.267 *p* = 0.12	r = −0.252 *p* = 0.144
AUD duration	r = 0.005 *p* = 0.972	r = −0.184 *p* = 0.243	r = −0.087 *p* = 0.62	r = −0.082 *p* = 0.642
Alcohol tolerance	r = −0.006 *p* = 0.968	r = −0.104 *p* = 0.51	r = 0.029 *p* = 0.868	r = −0.114 *p* = 0.514
Number heavy drinking days	r = 0.119 *p* = 0.442	r = 0.085 *p* = 0.593	r = 0.1 *p* = 0.575	r = −0.104 *p* = 0.557
Withdrawal duration	r = 0.117 *p* = 0.445	r = 0.035 *p* = 0.825	r = 0.246 *p* = 0.154	r = 0.042 *p* = 0.811
HDRS-17	r = −0.045 *p* = 0.767	r = −0.043 *p* = 0.784	r = −0.102 *p* = 0.561	r = 0.021 *p* = 0.903
AUDIT-C	r = 0.035 *p* = 0.82	r = −0.052 *p* = 0.74	r = −0.075 *p* = 0.669	r = −0.114 *p* = 0.513
OCDS	r = 0.072 *p* = 0.64	r = −0.019 *p* = 0.903	r = 0.203 *p* = 0.243	r = −0.052 *p* = 0.768
SHAPS	r = −0.046 *p* = 0.796	r = −0.183 *p* = 0.309	r = −0.195 *p* = 0.33	r = −0.355 *p* = 0.069

**Table 6:** Correlation analysis by Spearmen between β-endorphin and oxytocin level and aggressive patterns according to the BDHI in the two patient groups.

Buss–Durkee Index	AUD Group		AUD-MD Group	
	β-Endorphin	Oxytocin	β-Endorphin	Oxytocin
Physical aggression	r = −0.161 *p* = 0.354	r = −0.214 *p* = 0.225	r = −420 * ***p* = 0.026**	r = −0.293 *p* = 0.13
Indirect hostility	r = −0.243 *p* = 0.16	r = −0.211 *p* = 0.23	r = −0.122 *p* = 0.535	r = −0.001 *p* = 0.997
Irritability	r = −0.064 *p* = 0.713	r = −0.076 *p* = 0.671	r = −0.156 *p* = 0.429	r = −0.091 *p* = 0.645
Negativism	r = −0.045 *p* = 0.798	r = −0.102 *p* = 0.565	r = −0.114 *p* = 0.562	r = −0.039 *p* = 0.845
Resentment	r = −0.183 *p* = 0.292	r = −0.176 *p* = 0.32	r = −0.105 *p* = 0.593	r = −0.036 *p* = 0.855
Suspicion	r = −0.044 *p* = 0.803	r = −0.029 *p* = 0.872	r = 0.12 *p* = 0.542	r = 0.182 *p* = 0.353
Verbal hostility	r = −0.027 *p* = 0.88	r = −0.089 *p* = 0.617	r = −0.307 *p* = 0.112	r = −0.028 *p* = 0.887
Guilt	r = −0.179 *p* = 0.304	r = −0.111 *p* = 0.533	r = 0.152 *p* = 0.44	r = −0.063 *p* = 0.75

Note: * and in **bold**—significantly negatively correlated according to the Spearman test.

## Data Availability

The datasets generated for this study will not be made publicly available, but they are available on reasonable request to Svetlana A. Ivanova (ivanovaniipz@gmail.com), following approval of the Board of Directors of the MHRI, in line with local guidelines and regulations.
